# Untargeted metabolomics reveals stage-specific metabolic signatures in yak colostrum, transitional milk and mature milk

**DOI:** 10.1016/j.fochx.2025.102614

**Published:** 2025-06-02

**Authors:** Yunduan Wang, Daoliang Lan, Shaohui Beng, Shunyang Liu, Xueer Mu, Kechao Chen, Yueyue Li, Meng Xu, Jian Li, Wei Fu

**Affiliations:** a College of Animal & Veterinary Sciences, Southwest Minzu University, Chengdu 610041, China; b Key Laboratory of Qinghai-Tibetan Plateau Animal Genetic Resource Reservation and Utilization, Southwest Minzu University, Ministry of Education, Chengdu 610041, China; c Key Laboratory of Animal Science of National Ethnic Affairs Commission of China, Southwest Minzu University, Chengdu 610041, China

**Keywords:** Yak, Colostrum, Transitional milk, Mature milk, Metabolite

## Abstract

Yak milk's stage-specific metabolic profiles remain largely uncharacterized. In this study, yak colostrum (YC) was found to contain higher levels of essential components (e.g., milk fat, lactose, lactoprotein and non-fat solids) than transitional (YT) and mature milk (YM). A comprehensive untargeted profiling was conducted using UHPLC-Q Exactive HF-X to investigate the metabolic signatures of YC, YT and YM. A total of 2005 metabolites were identified. Metabolites enriched in YC were implicated in promoting intestinal microbiota colonization and supporting neonatal immune defense and neurodevelopment. In contrast, YM exhibited a metabolomic profile associated with sustained energy metabolism and the maturation of adaptive immune responses. YT exhibited a hybrid profile containing select bioactives characteristic of both YC and YM stages. Metabolites in YC and YT showed dynamic changes associated with circadian rhythm regulation and cognitive function under high-altitude hypoxia. This study reveals lactation-stage dynamics and supports functional development of yak milk products.

## Highlights

• 2005 stage-specific metabolites were identified in three stages of yak milk.
• Yak colostrum has higher level of sialylated oligosaccharides and choline.
• Yak colostrum and transitional milk implicate in circadian rhythm regulation.
• Yak milk support neurocognitive development in low-oxygen environment.

## Graphical abstract

**Figure:**
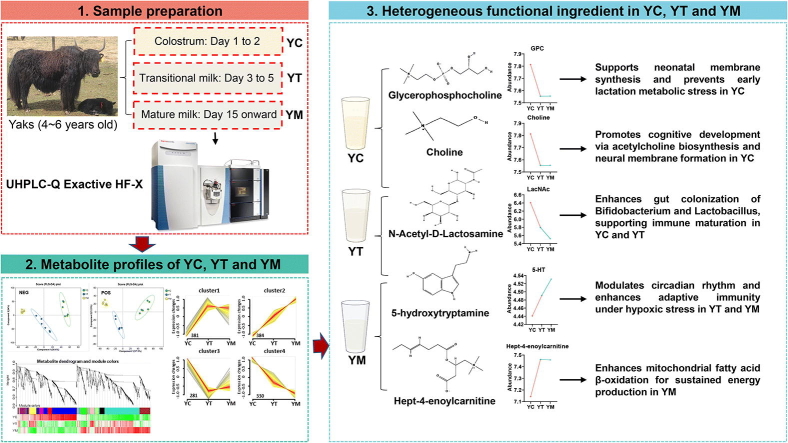

## 1 Introduction

Breast milk is widely recognized as the optimal natural source of infant nutrition, meeting all essential nutrient needs during early life and often referred to as the “gold standard” for neonatal nourishment (Scano et al., 2016; WHO, 2023). However, rapid economic development, urbanization, and various health challenges have significantly hindered breastfeeding practices, resulting in a global exclusive breastfeeding rate of less than 50 % among infants aged 0–6 months (Pérez-Escamilla et al., 2023). As a result, humanized infant formula has become an essential alternative, playing a critical role in supporting neonatal growth and development.

Milk plays a fundamental role in neonatal mammals by shaping the immune system, supporting brain development and cognitive function, and promoting the establishment of a healthy gut microbiota (Demmelmair et al., 2017; Maity & Ambatipudi, 2019; Nakajima et al., 2025). Milk can be broadly classified into colostrum, transitional milk, and mature milk. Colostrum is the first milk secreted during the first two days after birth (Arslan et al., 2021; Playford et al., 2000). It is particularly rich in growth factors and bioactive compounds that enhance intestinal barrier function and promote maturation of the neonatal digestive tract (Amarjeet et al., 2025; Lopez & Heinrichs, 2022). Additionally, colostrum contains immunoglobulin G (IgG) at concentrations nearly 100 times higher than those in mature milk (approximately 100 mg/mL), providing immediate passive immune protection and acting as the first line of defense for newborns (Korhonen et al., 2000; Ma et al., 2022). Transitional milk is secreted during the intermediate phase between colostrum and mature milk, typically from day 3 to 5 postpartum (Amarjeet et al., 2025; Godden, 2008). Mature milk refers to the milk produced approximately 2 to 3 weeks after parturition, typically beginning after day 14 postpartum (Bai et al., 2024; Chen et al., 2025).

The yak (*Bos grunniens*), a unique member of the Bovidae family, is a large ruminant well adapted to the extreme environmental conditions of high-altitude regions ranging from 3000 to 5000 m above sea level (Fan et al., 2020). In these regions, constant exposure to hypoxia, low temperatures, and intense ultraviolet radiation has driven the evolution of specialized physiological adaptations that enable yaks to survive under such extreme climatic conditions. These environmental pressures, combined with a natural grass-fed grazing system, have contributed to the exceptional nutritional composition of yak milk. Specifically, yak milk is rich in amino acids and their derivatives, contains elevated levels of lactose and vitamins, and provides a broad spectrum of unsaturated fatty acids (Chen et al., 2021; Kalwar et al., 2023; Li et al., 2024; Li, Liu, et al., 2023). Compared with camel, mare, and cow milk, yak milk is characterized by higher levels of fat, protein, and minerals (Kalwar et al., 2023). Compared with cow milk, yak milk contains approximately three times more protein and two to three times more fats (Lopez et al., 2017). Moreover, yak colostrum (YC) is enriched in α-linolenic acid (ALA) and docosahexaenoic acid (DHA), and contains higher levels of α-casein and whey proteins than cow milk. It also contains a higher lactose content than cow milk (Li et al., 2024; Luo et al., 2018; Playford & Weiser, 2021). In the harsh plateau environment, limited transportation and a scarcity of fruits and vegetables make yak milk and its dairy products an essential nutritional source for local herders (Guo et al., 2014; Kalwar et al., 2023; Li, Liu, et al., 2023). Remarkably, despite limited access to vitamins and minerals and extreme environmental stressors, local populations show notably low incidences of premature aging, edema, atherosclerosis, and cancer (Guo et al., 2014; Playford & Weiser, 2021; Wang et al., 2023). Furthermore, Ni et al. reported that mature yak milk shares neurodevelopmental regulatory and metabolic characteristics with human colostrum, highlighting its potential as an alternative lipid source in the formulation of human milk analogues (Ni et al., 2025). Therefore, elucidating the dynamic compositional changes in yak milk across lactation stages is essential for supporting its use as a functional ingredient in infant formula.

Metabolomics, a critical branch of omics sciences, plays a central role in characterizing the composition, relative abundance, dynamic changes, and interactions of metabolites in biological systems. Various metabolomic platform [e.g., nuclear magnetic resonance (NMR) spectroscopy, liquid chromatography–mass spectrometry (LC-MS), and gas chromatography–mass spectrometry (GC–MS)] have been extensively applied in dairy science research. In 2004, Chen et al. were the first to apply LC-MS technology to detect adulteration in goat milk (Chen et al., 2004). In 2010, Klein et al. utilized both NMR and GC–MS platforms to compare cow milk composition during early and late lactation, identifying 44 differentially expressed metabolites (DEMs) (Klein et al., 2010). More recently, Li et al. employed GC-TOF-MS to characterize 159 metabolites in human colostrum and mature milk, identifying 72 DEMs (Li et al., 2022). However, GC–MS was not applied to yak milk until 2017, when Chi et al. identified 21 volatile compounds (Chi et al., 2017). Li et al. further used GC–MS to demonstrate similar metabolic trends between yak and human colostrum and mature milk, reinforcing the potential of yak milk as a promising ingredient for infant formula (Li, Zeng, et al., 2023). In January 2025, Zhang et al. performed targeted metabolomic profiling and identified 362 metabolites in the colostrum of yak, buffalo, and Holstein cows (Zhang et al., 2025). They reported elevated concentrations of inositol, glycine, and carnitine in yak colostrum—nutrients known to support neonatal development. Despite growing interest in yak milk as a potential substitute for human breast milk due to its compositional similarity, the dynamic changes in its nutritional and bioactive components across lactation stages remain poorly understood.

In this study, UHPLC-Q Exactive HF-X was employed for the first time to perform comprehensive untargeted metabolomic profiling of YC, transitional milk (YT), and mature milk (YM). The aim was to systematically investigate metabolic pathways and their interactions across distinct lactation stages. In addition, time-series clustering analysis was conducted to reveal functional differences in metabolic processes related to immune regulation, gut microbiota colonization, and energy metabolism during yak lactation. This study provides novel insights into the dynamic compositional changes of yak milk, supports the development of yak-derived dairy innovations, and establishes a scientific basis for optimizing infant formula composition.

## 2 Materials and methods

### 2.1 Animal management and sample collection

Following Playford's classification of milk stages (Playford et al., 2000), yak colostrum (YC) was collected on days 1–2 postpartum, transitional milk (YT) on days 3–5, and mature milk (YM) from day 15 onward. All yaks used in this study were multiparous (4–6 years old, 260–330 kg) and were reared at approximately 4000 m altitude in Baiyu County, Garze Tibetan Autonomous Prefecture, Sichuan Province, under natural grazing without supplementary feeding. Milk samples were collected by hand milking and immediately stored at −80 °C. All animal experiments were conducted in compliance with the Regulations for the Administration of Experimental Animals, promulgated by the Ministry of Science and Technology of China (Beijing) in 1988 and last revised in 2017. All animal procedures were approved by the Institutional Animal Care and Use Committee of Southwest Minzu University (Approval Code: SMU-202401169).

### 2.2 Milk composition analysis

A LM2 Milk Analyzer (Master, Bulgaria) was used to quantify the contents of milk fat, lactose, lactoprotein, and non-fat solids in each sample. Each sample was analyzed in duplicate, and the average value was used for subsequent statistical analysis. The content percentages were calculated using the following formulas: milk fat (%) = (fat content / milk volume) × 100; lactose (%) = (lactose content / milk volume) × 100; lactoprotein (%) = (lactoprotein content / milk volume) × 100; non-fat solids (%) = (non-fat solids content / milk volume) × 100.

### 2.3 Sample preparation for non-targeted metabolomics

A total of 36 samples were collected, with 12 from each of the YC, YT, and YM groups. In each group, every two samples were pooled to generate one composite sample, yielding six replicates per group for further analysis. These samples were stored at −80 °C and sent to Majorbio (Shanghai, China) for UHPLC-Q Exactive HF-X analysis. All samples were thaw at 4 °C and homogenized before metabolite extraction. A total of 200 μL of yak milk was transferred into a 1.5 mL centrifuge tube, and 800 μL of extraction solvent [methanol:acetonitrile = 1: 1 (v: v)] containing 0.02 mg/mL of the internal standard L-2-chlorophenylalanine was added to extract the metabolites. The mixture was vortexed thoroughly for 30 s, followed by ultrasonic extraction at 5 °C and 40 kHz for 30 min to ensure efficient metabolite release. The samples were then kept at −20 °C for 30 min to precipitate proteins and other particulates. Afterward, they were centrifuged (13,000 ×*g*, 15 min, 4 °C). The supernatant was carefully collected and reconstituted in 120 μL of reconstitution solvent (acetonitrile: water = 1:1, v:v). The reconstituted samples were vortexed again for 30 s and underwent a second ultrasonic extraction under the same conditions (5 °C, 40 kHz) for 5 min to enhance solubility. Subsequently, the samples were centrifuged (13,000 ×*g*, 10 min, 4 °C). Finally, the supernatant was transferred to autosampler vials equipped with inserts for LC-MS analysis. Additionally, 20 μL aliquots of each sample supernatant were pooled to generate a quality control (QC) sample for system stability monitoring.

### 2.4 UHPLC-Q Exactive HF-X analysis

Chromatographic separation was performed using an ACQUITY UPLC HSS T3 column (100 mm × 2.1 mm, 1.8 μm, Waters, Milford, USA) coupled to a UHPLC-Q Exactive HF-X system (Thermo Fisher Scientific, MA, USA), with a 3 μL injection volume per sample. Mobile phase A consisted of 95 % water and 5 % acetonitrile (*v*/v), containing 0.1 % formic acid, while mobile phase B comprised 47.5 % acetonitrile, 47.5 % isopropanol, and 5 % water (v/v/v), also with 0.1 % formic acid.

Mass spectrometric data were acquired on a Q Exactive HF-X instrument (Thermo Fisher Scientific, MA, USA) equipped with an electrospray ionization (ESI) source operating in both positive and negative ion modes. The optimized ESI source parameters were set as follows: sheath gas flow rate, 50 arb; auxiliary gas flow rate, 13 arb; auxiliary gas heater temperature, 425 °C; capillary temperature, 325 °C. The ion spray voltage floating (ISVF) was set to +3500 V in positive ion mode and − 3500 V in negative ion mode. Normalized collision energy (NCE) was applied in stepped mode at 20, 40, and 60 eV to ensure broad coverage of fragment ion spectra. The resolution was set to 60,000 (at *m*/*z* 200) for full MS scans and 7500 (at m/z 200) for MS/MS scans. To ensure data quality and analytical reproducibility, pooled QC samples were injected at regular intervals (every 5–15 samples) to monitor instrument stability and performance throughout the run.

### 2.5 UHPLC-Q Exactive HF-X data analysis

The raw mass spectrometry data were imported into Progenesis QI software (version 3.0, Waters Corporation, Milford, USA) for preprocessing, including baseline correction, peak detection, integration, retention time alignment, and peak matching. Metabolite identification was performed by matching MS and MS/MS spectral data against the Human Metabolome Database (HMDB) and METLIN database, using a mass accuracy tolerance of less than 10 ppm. Metabolite annotation was further confirmed based on MS/MS fragmentation pattern matching scores. Following database searching, missing values in the data matrix were filtered using the 80 % rule, and missing data were imputed. Peak intensities were normalized using the total ion current (TIC) method to minimize technical variation and obtain a normalized data matrix. Variables with a relative standard deviation (RSD) exceeding 30 % in quality control (QC) samples were excluded from further analysis. The processed data were subsequently subjected to log10 transformation prior to statistical analysis.

Multivariate statistical analyses, including partial least squares discriminant analysis (PLS-DA) and orthogonal partial least squares discriminant analysis (OPLS-DA), were conducted using the R package “ropls” (version 1.6.2). Differentially expressed metabolites (DEMs) were identified based on variable importance in projection (VIP) scores derived from the OPLS-DA model and *p*-values obtained from Student's *t*-test. Metabolites with a *p*-value <0.05 and VIP value ≥1 were considered DEMs between the two groups.

### 2.6 Bioinformatics analysis

Bioinformatic analyses of UHPLC-Q Exactive HF-X dataset were conducted using the Majorbio Cloud platform (https://cloud.majorbio.com). Functional annotation of yak milk metabolites was carried out using Kyoto Encyclopedia of Genes and Genomes (KEGG) (http://www.genome.jp/kegg/). Python (Version1.0.0) software package scipy.stats was used for pathway enrichment analysis (*P* < 0.05). Clustering analysis was performed with the Mfuzz package (version 2.6.0, https://www.bioconductor.org/packages/release/bioc/html/Mfuzz.html). The number of clusters was set as 6. Weighted Gene Co-expression Network Analysis (WGCNA) analysis was performed with the R software package wgcna (Version 1.6.8). All data visualization steps were carried out in GraphPad Prism 8.0.2 (GraphPad, Santiago, USA).

## 3 Results and discussion

### 3.1 Identification of the metabolite composition in yak colostrum (YC), transitional milk (YT) and mature milk (YM)

**Fig. 1:**
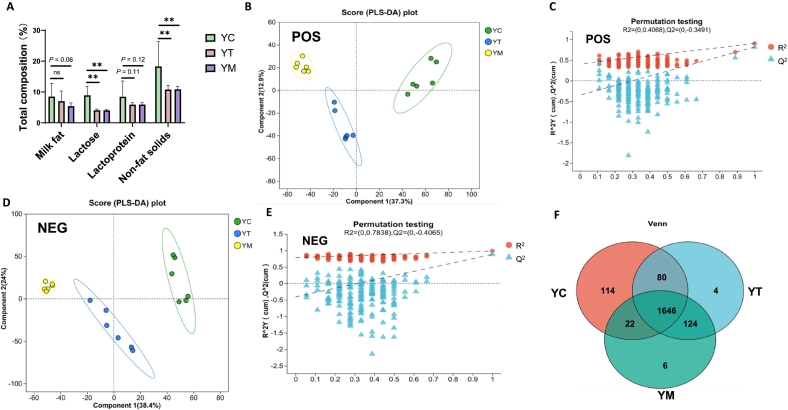
Characterization of Metabolite Composition in yak colostrum (YC), transitional milk (YT) and mature milk (YM). (A) Characterization of yak milk across different lactation stages.  *n* = 12. (B—C) Partial least squares discriminant analysis (PLS-DA) of scores (B) and permutation test plots (*200 times*) (C) for the YC, YT and YM analyzed in the positive ion mode. (D-E) PLS-DA of scores (D) and permutation test plots (200 times) (E) for the YC, YT and YM analyzed in the negative ion mode. (F) Venn diagram of the identified yak milk metabolites. *n* = 6; *p < 0.05, **p < 0.01, ns represents no significance.

Colostrum, the initial secretion from the mammary glands after parturition, is rich in nutrients and bioactive compounds. It serves as the primary nutritional source for neonatal mammals, playing a critical role in supporting early growth, development, and immune protection. At parturition, yak colostrum (YC) contained 8.48 % milk fat, 8.90 % lactose, 8.43 % lactoprotein, and 18.28 % non-fat solids (Fig. 1A). In transitional milk (YT), these levels decreased to 7.00 %, 4.06 %, 5.90 %, and 10.80 %, respectively, marking the beginning of the transition toward mature milk (Fig. 1A). In mature milk (YM), the respective values further decreased to 5.38 %, 4.05 %, 5.92 %, and 10.87 % (Fig. 1A). As shown in Fig. 1A, YC exhibited significantly higher levels of lactose (*P* < 0.01) and non-fat solids (*P* < 0.01) compared with both YT and YM. Milk fat content in YC was higher than in YM (*P* = 0.06), whereas no significant difference was detected between YC and YT. Although lactoprotein content showed a decreasing trend from YC to YM, the differences were not statistically significant (*P* = 0.12 between YC and YT; *P* = 0.11 between YC and YM). These findings suggest that the most pronounced compositional changes during early lactation occur in lactose and non-fat solids, potentially reflecting alterations in energy and nutrient provisioning strategies. The composition and physicochemical properties of colostrum are influenced by factors such as breed, individual variation, age, and nutritional status. Compared with mature milk, colostrum contains higher concentrations of fat, protein, vitamins, minerals, cytokines, and growth factors (Linehan et al., 2023). These bioactive components decline substantially within the first three days postpartum, as the milk transitions toward the nutrient profile characteristic of mature milk (Playford & Weiser, 2021).

To comprehensively elucidate the metabolic differences in yak milk across distinct lactation stages, an untargeted metabolomics approach was employed to systematically profile metabolite composition and variation. Partial least squares discriminant analysis (PLS-DA) revealed distinct metabolic profiles among YC, YT, and YM under both positive (POS) and negative (NEG) ion modes (Fig. 1B and D). Each group, comprising six biological replicates, exhibited clear clustering in the PLS-DA score plots under both ion modes, indicating high reproducibility and low intra-group variability. Model parameters, including R^2^ and Q^2^, were within acceptable confidence intervals, supporting the predictive accuracy, robustness, and stability of the PLS-DA model (Fig. 1C and E). Hierarchical clustering results were consistent with the PLS-DA findings, jointly confirming distinct intergroup separation and supporting data robustness and reliability (Fig. S1A). A total of 10,140 chromatographic peaks were detected across both ionization modes in the untargeted metabolomics dataset. After excluding features with more than 20 % missing values and ensuring that the relative standard deviation (RSD) of quality control (QC) samples remained below 30 %, 2005 metabolites were successfully annotated, including 1213 in POS and 792 in NEG mode (Table S1). Of the 2005 annotated metabolites, 392 were mapped to the KEGG pathway database. Of these, 382 were linked to specific metabolic pathways, including 92 involved in lipid metabolism, 43 in nucleotide metabolism, and 111 in amino acid metabolism (Fig. S1B). Annotation using the Human Metabolome Database (HMDB) identified 1666 metabolites, which were classified into 15 distinct superclasses. Among these, lipids and lipid-like molecules (501), organic oxygen compounds (277), and organophosphorus compounds (273) were the most abundant, together accounting for 36.09 % of all annotated metabolites (Fig. S1C). The Venn diagram is based on 2005 metabolites  (Fig. 1F).

### 3.2 Analysis of differentially expressed metabolites (DEMs) in yak milk among different lactation periods

A supervised orthogonal partial least squares discriminant analysis (OPLS-DA) model was utilized to analyze the data. This model enhances interpretability by removing variables with low correlation to the Y component and reducing model complexity without compromising predictive accuracy (Lu et al., 2024). In this study, the OPLS-DA model effectively discriminated yak milk samples from different lactation stages and identified differentially expressed metabolites. In both POS and NEG ion modes, a clear separation was observed between the YC and YM groups (Fig. S2A). Permutation tests (*n* = 200) confirmed the robustness of the model, with cumulative explanatory (R^2^Y) and predictive (Q^2^) values indicating excellent model fit and strong predictive ability (Fig. S3A). Similarly, comparisons between YT vs. YM and YC vs. YT yielded well-fitted OPLS-DA models with no evidence of overfitting (Fig. S2B-C and Fig. S3B—C). Variable importance in projection (VIP) scores derived from the OPLS-DA model were used as the primary metric for identifying potential metabolic biomarkers. DEMs were selected based on the criteria of *P* < 0.05 and VIP > 1.

**Fig. 2:**
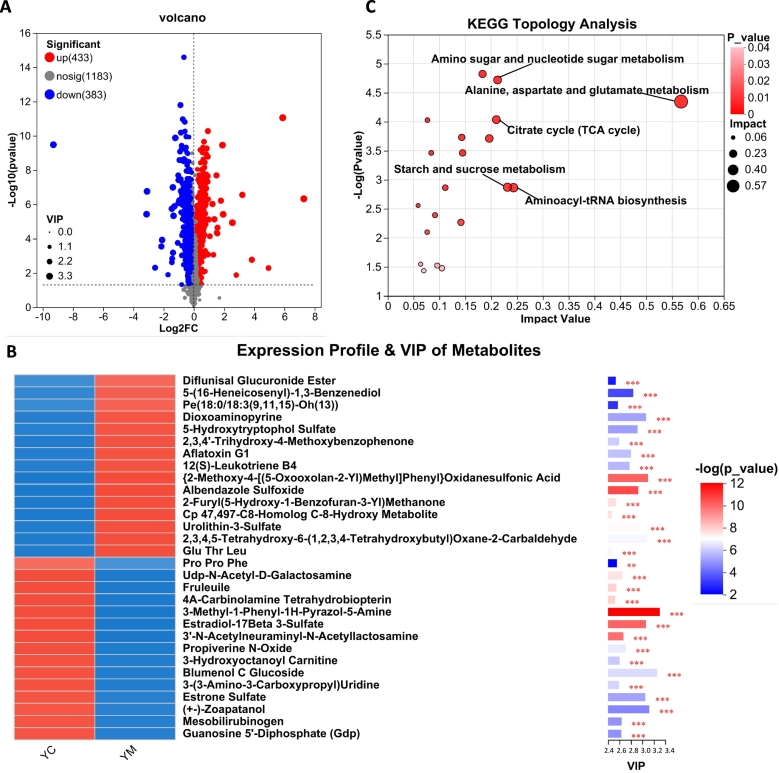
Differentially expressed metabolites (DEMs) analysis between YC and YM. (A) Volcano plot of DEMs between YC and YM. Red dots represent upregulated metabolites in YC relative to YM, blue dots represent downregulated metabolites, and gray dots indicate metabolites with no significant difference. (B) Metabolite clustering heat map analysis and variable importance in projection (VIP) scores of DEMs between YC and YM. VIP score was based on the OPLS-DA model. Significant differences were compared with each two groups. (C) Metabolome view map of the significant metabolic pathways characterized between YC and YM. The circles represent the KEGG enriched pathways of differential metabolites (*p* <0.05). Darker colors and larger sizes respectively indicate higher pathway enrichment and greater pathway impact values. *n* = 6; *p < 0.05, **p < 0.01, ***p < 0.001. (For interpretation of the references to colour in this figure legend, the reader is referred to the web version of this article.)

(1) A total of 816 DEMs were identified in YC compared with YM, including 433 upregulated and 383 downregulated metabolites (Fig. 2A and Table S2). These DEMs were ranked by VIP scores, and the top 30 metabolites were displayed in a heatmap (Fig. 2B). We first examined the metabolites that were significantly upregulated in YC compared with YM. Estradiol-17β 3-sulfate (a sulfated form of estradiol) and estrone sulfate (a sulfated form of estrone) are major estrogen derivatives. Estrone sulfate can be converted into its more active form, estradiol, through the action of steroid sulfatase (STS) (Mueller et al., 2015). The sustained elevation of conjugated estrogens (e.g., estrone sulfate and estradiol sulfate) immediately after parturition reflects delayed metabolic clearance of hormones accumulated during pregnancy (Stanczyk, 2024). This postpartum hormonal pattern is conserved among mammalian species. Similar observations in humans and dairy cattle underscore a conserved adaptive strategy during early lactation (Heap & Hamon, 1979; Tulchinsky et al., 1972). Moreover, estradiol activates the PI3K/AKT/mTOR signaling cascade, promoting G1-to-S phase transition and facilitating G2/M progression, thereby stimulating cell proliferation (Zhou et al., 2024). Activation of mTOR enhances protein biosynthesis in skeletal muscle and liver, indirectly influencing amino acid metabolism (Saxton & Sabatini, 2017). 3-(3-Amino-3-carboxypropyl) uridine, a modified nucleoside found in tRNA, plays a critical role in enhancing translational efficiency and fidelity (Takakura et al., 2019). UDP-*N*-acetyl-D-galactosamine (UDP-GalNAc) and 3’-*N*-acetylneuraminyl-*N*-acetyllactosamine are essential intermediates in glycosylation pathways. Specifically, the terminal sialylation of 3’-*N*-acetylneuraminyl-*N*-acetyllactosamine depends on prior GalNAc addition, catalyzed by glycosyltransferases that utilize UDP-GalNAc as a donor substrate (Sasaki et al., 1997). Importantly, 3’-*N*-acetylneuraminyl-*N*-acetyllactosamine, a sialylated oligosaccharide, plays a vital role in supporting infant growth and development by promoting neurodevelopment, maintaining cognitive function, and enhancing immune responses (Zhang et al., 2019). Moreover, sialylated oligosaccharides promote the colonization of beneficial gut microbiota and inhibit pathogen adhesion to the intestinal epithelium, thereby enhancing mucosal antiviral defense in neonates (Gnoth et al., 2000). 3-Hydroxyoctanoyl carnitine facilitates the transport of fatty acids into mitochondria, where they undergo β-oxidation, resulting in energy production and the generation of intermediates such as guanosine 5′-diphosphate (Hanley et al., 2005). These DEMs were notably upregulated in YC compared to YM. In contrast, several DEMs were significantly downregulated, as detailed below. 5-Hydroxytryptophol (5-HTOL) is sulfated by sulfotransferases to produce 5-HTOL sulfate, the terminal metabolite of 5-hydroxytryptamine (5-HT) catabolism (Liu et al., 2021). Notably, 5-HT, one of the three principal tryptophan (Trp) metabolites, plays a pivotal role in regulating systemic inflammation, intestinal motility, gastrointestinal secretion, and nutrient absorption (Agus et al., 2018; Mawe & Hoffman, 2013). These findings suggest that YM may promote gastrointestinal motility in newborns, facilitate digestion, and enhance nutrient absorption. 12(*S*)-Leukotriene B4, a biologically active derivative of leukotriene B4 (LTB4), functions as a potent pro-inflammatory mediator derived from arachidonic acid metabolism. It acts synergistically with TNF-α and IL-1, thereby contributing to tissue repair and immune surveillance (Yokomizo, 2015). Phosphatidylethanolamine (PE) undergoes hydroxylation to form PE [18:0/18:3(9,11,15)-OH(13)]. In milk, PE is readily absorbed by neonates and plays crucial roles in brain phospholipid biosynthesis and energy metabolism (Nilsson et al., 2021). Compared with YC, YM provides a more sustained energy supply and supports continuous neonatal growth. It is speculated that during the YC stage, sialylated oligosaccharides promote the colonization of beneficial microbes (e.g., bifidobacteria) in the infant gut, thereby forming an anti-inflammatory barrier and supporting immune system development. Over time, local inflammatory mediators (e.g., LTB4) may become more abundant and readily detectable during the mature milk phase.

KEGG pathway enrichment analysis identified five major enriched pathways: amino sugar and nucleotide sugar metabolism, alanine, aspartate and glutamate metabolism, citrate cycle (TCA cycle), starch and sucrose metabolism, and aminoacyl-tRNA biosynthesis (Fig. 2C). These enriched pathways reflect distinct metabolic adaptations between YC and YM, underscoring their specialized roles in immune protection and nutritional support during different lactation stages. Colostrum is particularly rich in proteins (e.g., immunoglobulins, bioactive peptides, antioxidants, and functional oligosaccharides), which collectively contribute to passive immunity acquisition (Lopez & Heinrichs, 2022). During the YC stage, enhanced protein synthesis supports early immune and neurological development in infants (Lopez & Heinrichs, 2022). Accordingly, pronounced enrichment was observed in pathways related to amino sugar and nucleotide sugar metabolism (critical for the biosynthesis of oligosaccharides and glycoproteins), alanine, aspartate, and glutamate metabolism (essential for the synthesis of immunoproteins and bioactive peptides), and aminoacyl-tRNA biosynthesis (which supports rapid protein production during early neonatal development) (Fig. 2C). As neonates gradually adapt to the external environment and establish more robust endogenous immune systems, metabolic priorities shift toward meeting the increasing demands for sustained macronutrient supply, particularly lactose and lipids. At the YM stage, the TCA cycle becomes central to ATP production, while starch and sucrose metabolism provide essential precursors for lactose synthesis. Both pathways become increasingly important in meeting the energy demands and growth requirements during late lactation.

To further highlight the uniqueness of yak milk, we compared the key metabolites and metabolic pathways identified in YC and YM with those reported in human breast milk. According to Li et al. (Li et al., 2022), a metabolomics-based comparison between human colostrum and mature milk revealed 72 DEMs, most of which were involved in amino acid metabolism (e.g., glycine, serine, and threonine metabolism), organic acid metabolism (e.g., glyoxylate and dicarboxylate metabolism), and energy production pathways (e.g., alanine, aspartate, and glutamate metabolism). These findings are consistent with our KEGG enrichment results, which showed that YC is markedly enriched in amino sugar and nucleotide sugar metabolism as well as aminoacyl-tRNA biosynthesis, both essential for early protein synthesis and immune development. Moreover, KEGG enrichment of human-specific metabolites highlighted pathways such as galactose metabolism, glutathione metabolism, and linoleic acid metabolism, which are associated with redox balance and mucosal immunity. In contrast, yak-specific metabolites were enriched in the pentose phosphate pathway and aminoacyl-tRNA biosynthesis, implicating elevated antioxidant capacity and enhanced protein synthesis in early neonatal adaptation. These findings underscore the adaptive metabolic programming of yak milk, especially its colostrum, to support offspring survival in high-altitude hypoxic environments.

However, YC exhibited a metabolite profile distinct from that of human colostrum. For instance, sialylated oligosaccharides such as 3’-*N*-acetylneuraminyl-*N*-acetyllactosamine were significantly enriched in YC, yet were absent from the major DEMs identified in human milk. Furthermore, human mature milk contains elevated levels of glutaric acid and prostaglandin E2 (PGE2), both of which are involved in fatty acid metabolism and immune modulation. In contrast, YM was enriched in energy-generating metabolites, including 3-hydroxyoctanoyl carnitine and intermediates of the TCA cycle, indicating species-specific adaptations in energy supply strategies during late lactation. Taken together, both human and yak milk exhibit dynamic metabolomic reprogramming across lactation stages. However, yak milk appears to prioritize amino acid metabolism, sialylation, and mitochondrial energy production, possibly reflecting evolutionary adaptation to high-altitude environments. These comparative findings strengthen the rationale for considering yak milk as a promising functional ingredient in infant formula designed to support immunological and metabolic development.

**Fig. 3:**
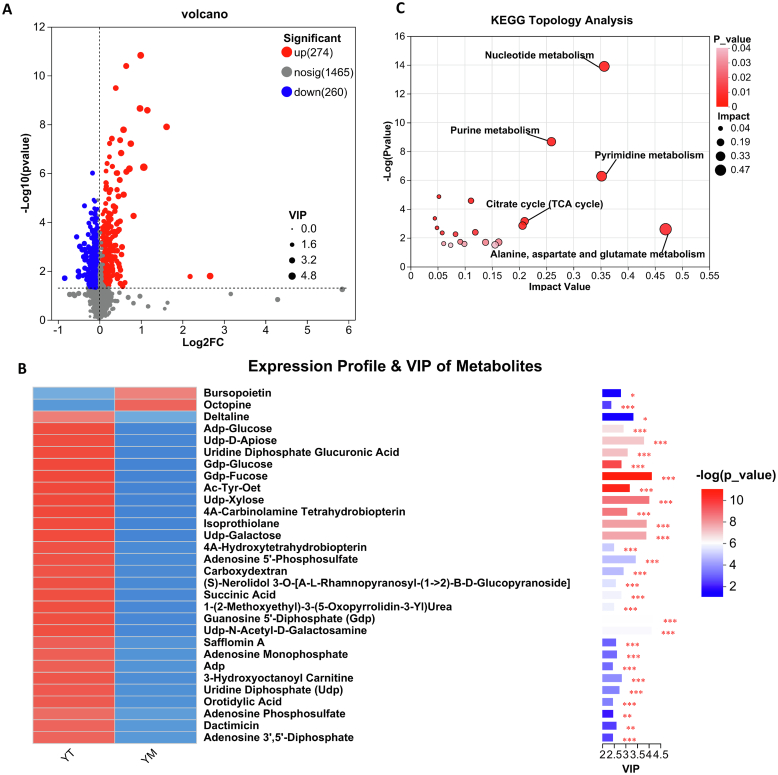
DEMs analysis between YT and YM. (A) Volcano plot of DEMs between YT and YM. Red dots represent upregulated metabolites in YT relative to YM, blue dots represent downregulated metabolites, and gray dots indicate metabolites with no significant difference. (B) Metabolite clustering heat map analysis and VIP scores of DEMs between YT and YM. VIP score was based on the OPLS-DA model. Significant differences were compared with each two groups. (C) Metabolome view map of the significant metabolic pathways characterized between YT and YM. The circles represent the KEGG enriched pathways of differential metabolites (*p* <0.05). Darker colors and larger sizes respectively indicate higher pathway enrichment and greater pathway impact values. *n* = 6; *p < 0.05, **p < 0.01, ***p < 0.001. (For interpretation of the references to colour in this figure legend, the reader is referred to the web version of this article.)
(3) DEMs analysis between YC and YT: A total of 782 DEMs were identified between YC and YT, including 411 upregulated and 371 downregulated metabolites in YC relative to YT (Fig. 4A and Table S4). We first examined the metabolites that were significantly upregulated in YC compared with YT. Among the top 30 DEMs ranked by VIP scores were estrogen derivatives (e.g., estrone and estradiol sulfate), tRNA-modified nucleosides [e.g., 3-(3-amino-3-carboxypropyl) uridine), and sialylated oligosaccharides (e.g., 3’-*N*-acetylneuraminyl-*N*-acetyllactosamine) (Fig. 4B). Together, these metabolites underscore the multifaceted roles of YC in regulating neonatal endocrine function, enhancing innate immunity, and promoting gastrointestinal maturation and physiological adaptation. The downregulated DEMs included 5-HTOL sulfate, 12(*S*)-leukotriene B4, and phosphatidylethanolamine [PE(18,4/0,0)]. These findings suggest that YT no longer prioritizes immune protection to the same extent as YC, nor has it fully acquired the energy-dense nutritional profile characteristic of YM. Instead, YT represents an intermediate stage that balances neonatal immune support with increasing nutrient and energy delivery. KEGG pathway enrichment analysis revealed significant enrichment of the aminoacyl-tRNA biosynthesis pathway, accompanied by elevated levels of essential amino acids such as L-phenylalanine (Phe) and L-tyrosine (Tyr) (Fig. 4C). As these amino acids cannot be synthesized de novo by neonatal mammals, dietary intake directly influences the efficiency of protein biosynthesis (Kilberg et al., 2005). L-Lysine (Lys) is essential not only for polypeptide chain elongation during translation but also as a key residue for post-translational modifications, including acetylation, methylation, and ubiquitination (Yang & Seto, 2008). Additionally, Phe and Tyr play indispensable roles in stabilizing enzyme active sites, maintaining hydrophobic core structures, and mediating cell signaling events, particularly through Tyr phosphorylation (Schlessinger, 2000). These three upregulated DEMs indicate that YC promotes a higher rate of protein synthesis and post-translational modification compared with YT. Within the Riboflavin metabolism pathway, elevated levels of free Riboflavin, the primary bioactive form of vitamin B_2_, promote enhanced intestinal absorption and antioxidant capacity. Conversely, flavin mononucleotide (FMN), a phosphorylated derivative of riboflavin, was detected at reduced levels. FMN primarily functions as a coenzyme in mitochondrial electron transport and cellular energy metabolism (Barile et al., 2016). During the YC stage, maternal secretion of free riboflavin enhances neonatal antioxidant defenses and facilitates rapid immune modulation. As lactation progresses, YT exhibits a marked increase in energy and nutrient supply, as evidenced by the enrichment of starch and sucrose metabolism pathways. In summary, both YC and YT contribute to enhanced immune responses by promoting the colonization of beneficial gut microbes. However, YC exhibits a higher rate of protein synthesis, potentially reflecting the neonatal requirement to adapt to the external environment by increasing levels of immunoglobulins, bioactive peptides, and functional oligosaccharides at birth. While maintaining immunological support, YT progressively shifts toward enhancing energy and lactose delivery to sustain neonatal growth and metabolic development.

(2) DEMs analysis between YT and YM: A total of 534 DEMs were identified, including 274 upregulated and 260 downregulated metabolites in YT relative to YM (Fig. 3A and Table S3). We first examined metabolites that were significantly upregulated in YT compared with YM. Among the top 30 DEMs ranked by VIP scores (Fig. 3B), guanosine 5′-diphosphate (GDP), uridine diphosphate (UDP), adenosine diphosphate (ADP), adenosine monophosphate (AMP), orotidylic acid (OMP), GDP-fucose, UDP-GalNAc, and UDP-Gal were closely associated with nucleotide sugar biosynthesis and cellular energy metabolism. GDP, ADP, AMP, and UDP function as key intermediates in both cellular energy metabolism and the biosynthesis of purine and pyrimidine nucleotides. In contrast, OMP is specific to the pyrimidine biosynthetic pathway and acts as a precursor for nucleotide sugar formation and RNA synthesis. Additionally, high-energy phosphate bonds from ATP and GTP are required to drive the synthesis of UDP- and GDP-linked sugars, which in turn fuel protein translation, secretion, and other energy-intensive cellular processes (Lane & Fan, 2015). Both UDP-GalNAc and UDP-Gal share a UDP backbone linked to distinct monosaccharides, forming high-energy nucleotide sugars that function as glycosyl donors in the glycosylation of proteins, lipids, and oligosaccharides. Notably, UDP-Gal functions as a galactose donor in lactose biosynthesis, whereas UDP-GalNAc initiates mucin-type O-glycosylation, a process essential for the formation of the mucosal barrier (Holden et al., 2003; McGuckin et al., 2011). Mucin-type O-glycosylation promotes the development of a mucus layer enriched with immune components (e.g., IgA, complement proteins and antimicrobial peptides), thereby preventing pathogen invasion and adhesion (McGuckin et al., 2011). Furthermore, this mucus layer modulates cellular signaling and inflammatory responses while facilitating the colonization of commensal probiotic bacteria, thereby contributing to intestinal microbial homeostasis (Schroeder, 2019). 4a-Carbinolamine and 4a-hydroxytetrahydrobiopterin are oxidative intermediates of tetrahydrobiopterin (BH4), representing distinct structural forms within the same oxidation pathway. These intermediates are commonly referred to as “4a-hydroxy-BH4 derivatives” in the literature (Thöny et al., 2000). Maintaining dynamic balance within the BH4 cycle is essential for the synthesis of key neurotransmitters, including dopamine, serotonin, norepinephrine, and nitric oxide. This balance plays a critical role in regulating neurological, cardiovascular, and immune functions (Werner et al., 2011). Adenosine phosphosulfate (APS) is a central intermediate in the sulfation pathway, which is essential for detoxification, hormone regulation, and drug metabolism (Strott, 2002). KEGG pathway enrichment analysis revealed that metabolic pathways related to nucleotide metabolism, specifically purine and pyrimidine metabolism, were among the most significantly enriched (Fig. 3C). As lactation progresses, the demand for nucleotide precursors in both neonates and mothers remains high or may even increase, supporting extensive DNA and RNA biosynthesis required for rapid neonatal growth. YT exhibits a hybrid metabolic profile that integrates the robust immune protection and abundance of bioactive molecules characteristic of colostrum with the enhanced energy and nutrient supply observed in YM. At the metabolomic level, this transition is characterized by significant enrichment in pathways related to nucleotide biosynthesis, amino acid metabolism, and the TCA cycle.

**Fig. 4:**
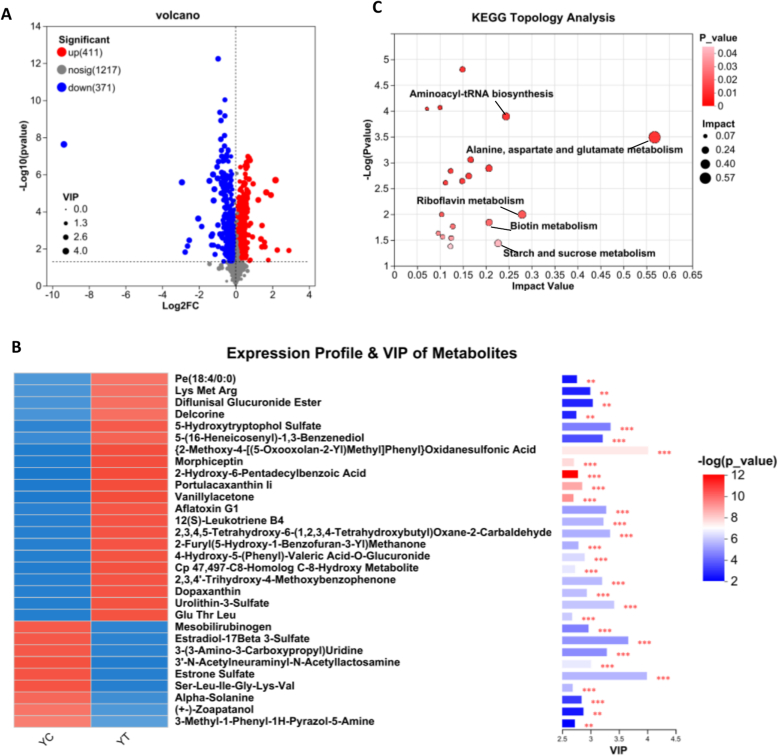
DEMs analysis between YC and YT. (A) Volcano plot of DEMs between YC and YT. Red dots represent upregulated metabolites in YC relative to YT, blue dots represent downregulated metabolites, and gray dots indicate metabolites with no significant difference. (B) Metabolite clustering heat map analysis and VIP scores of DEMs between YC and YT. VIP score was based on the OPLS-DA model. Significant differences were compared with each two groups. (C) Metabolome view map of the significant metabolic pathways characterized between YC and YT. The circles represent the KEGG enriched pathways of differential metabolites (*p* <0.05). Darker colors and larger sizes respectively indicate higher pathway enrichment and greater pathway impact values. *n* = 6; *p < 0.05, **p < 0.01, ***p < 0.001. (For interpretation of the references to colour in this figure legend, the reader is referred to the web version of this article.)

### 3.3 Heterogeneous functions of milk metabolites at the processes of yak lactation

**Fig. 5:**
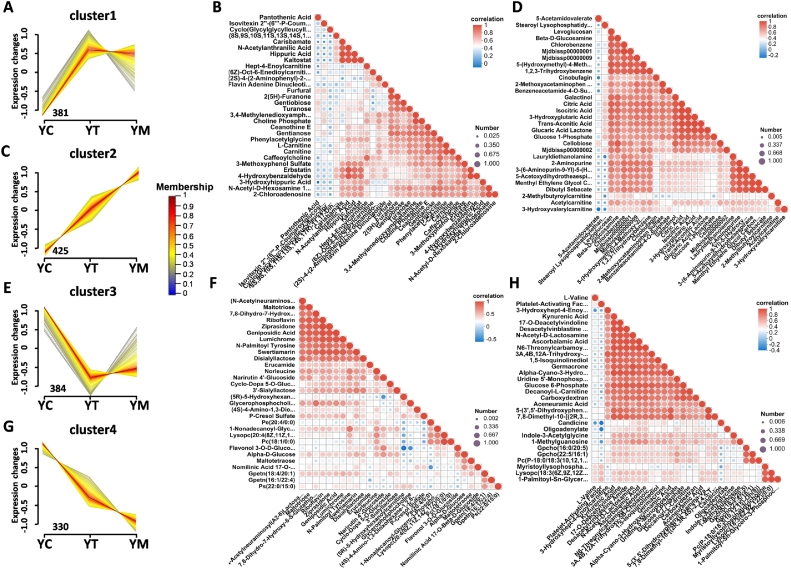
Heterogeneous functions of the Metabolite in YC, YT and YM. (A, C, E and G) Mfuzz time-series clustering analysis showing expression trends of metabolites across three stages. (B, D, F and H) Correlation heat map colour-coded by the strength of Pearson correlation matrix among the Top 30 metabolites in cluster1 (B), cluster2 (D), cluster3 (F) and cluster4 (H). Red boxes indicate positive association; blue boxes indicate negative association. (For interpretation of the references to colour in this figure legend, the reader is referred to the web version of this article.)

Subsequently, Mfuzz time-series clustering analysis was performed to examine the dynamic variation in metabolite expression across distinct lactation stages (Table S5). Cluster 1, containing 381 metabolites, was characterized by low expression in YC, followed by consistently elevated levels in YT and YM (Fig. 5A and Table S5). KEGG pathway enrichment analysis showed that these metabolites were significantly associated with pathways including vitamin digestion and absorption, cofactor biosynthesis, and regulation of lipolysis in adipocytes (Fig. S4A). These pathways are closely involved in lipid metabolism, particularly lipid degradation, which provides a substantial energy supply. Among Cluster 1 metabolites, the top 30 most strongly correlated included L-carnitine, which facilitates the transport of fatty acids into mitochondria, where they are converted into acylcarnitines [e.g., (6Z)-oct-6-enoylcarnitine and hept-4-enoylcarnitine] (Fig. 5B). These intermediates subsequently undergo β-oxidation, a process that requires FAD as a cofactor and results in the production of coenzyme A (CoA), which enters the TCA cycle for energy generation (Fig. 5B) (Xiang et al., 2025). Notably, pantothenic acid acts as a direct precursor for CoA synthesis. 2(5H)-furanone, an oxidative metabolite of linoleic acid, was identified in this cluster (Liu et al., 2019). Furthermore, choline phosphate and caffeoylcholine contribute to neural regulation and antioxidant defense, respectively (Gülçin, 2006; Zeisel & da Costa, 2009). The biosynthesis of these compounds depends on amino acids such as methionine, serine, Phe, and Tyr. KEGG enrichment analysis indicated that these metabolic pathways (e.g., Phenylalanine, tyrosine and tryptophan biosynthesis) were significantly enriched (Fig. S4A).

Cluster 2 comprises 425 metabolites that exhibit a progressive increase from YC through YT to YM (Fig. 5C and Table S5). In the metabolite correlation heatmap, Glucose 1-Phosphate contributes directly to the production of acetyl coenzyme A (acetyl-CoA) through glycolysis, similar to carnitine-derived metabolites (e.g., 2-methylbutyrylcarnitine, acetylcarnitine, and 3-hydroxyvalerylcarnitine) (Fig. 5D) (Li et al., 2025; Minkler et al., 1990). The resulting acetyl-CoA subsequently enters the TCA cycle, where it is converted into key intermediates such as citric acid, isocitric acid, and trans-aconitic acid, ultimately supporting ATP production (Ly et al., 2020; Xiang et al., 2025) (Fig. 5D). KEGG enrichment analysis revealed significant enrichment in metabolic pathways related to the TCA cycle and glycolysis, including TCA cycle, biosynthesis of cofactors, and central carbon metabolism in cancer (Fig. S4B). In addition, pathways related to amino acid metabolism and the pentose phosphate pathway, such as vitamin B6 metabolism, glutamatergic synapse, alanine, aspartate, and glutamate metabolism, were also significantly enriched. Glutamine serves as a precursor for arginine biosynthesis, an essential amino acid that is often limited in colostrum. Notably, arginine has been shown to attenuate hypoxia-induced upregulation of hypoxia-inducible factor 1-alpha (HIF-1α) mRNA expression, thereby enhancing resistance to hypoxic stress (Varghese et al., 2022; Wu & Knabe, 1995). Similarly, hypoxia is closely associated with oxidative stress, whereas the pentose phosphate pathway plays a crucial role in mitigating oxidative damage and enhancing neonatal brain resilience (Brekke et al., 2014). Based on these findings, we propose that yak milk may facilitate hypoxia adaptation by promoting arginine biosynthesis. In YC, predominant metabolites are primarily involved in immune modulation, supporting the establishment of early immune defenses in neonates. Given that yaks inhabit cold, high-altitude environments, the metabolic profile during the YT and YM stages shifts toward comprehensive nutrient provision and enhanced hypoxia tolerance. This metabolic adaptation supports the increasing demands of neurodevelopment and somatic growth in newborn calves. Such adaptation is essential for maintaining neurocognitive function and brain development in neonatal calves inhabiting the hypoxic environment of high-altitude plateaus.

Cluster 3, comprising 384 metabolites, is characterized by high expression levels in YC, followed by consistently lower expression in YT and YM (Fig. 5E and Table S5). Notably, these metabolites were significantly enriched in the bile secretion pathway (Fig. S4C). Choline, a key metabolite in these pathways, was highly enriched in YC. Consistent with these findings, elevated choline levels were also observed in the colostrum of Ladakhi cows (Amarjeet et al., 2025). Choline functions as a critical precursor for membrane phospholipid biosynthesis, which is essential for the rapid proliferation of neonatal cells immediately after birth (van der Veen et al., 2017). Additionally, choline contributes to cognitive development through its involvement in the synthesis of the neurotransmitter acetylcholine (Zeisel, 2000). Previous studies have reported high concentrations of choline metabolites in bovine colostrum, suggesting their potential as functional bioresources for infant formula supplementation (Bernhard et al., 2020). Furthermore, strong correlations were observed among glycerophospholipid (GPL)-associated metabolites, including PE(20:4/0:0), PS(22:0/15:0), GPETN(16,1/22:4), PC(18,1/0,0), and glycerophosphocholine (GPC). GPL play a fundamental role in constructing the bilayer architecture of cellular membranes, maintaining membrane fluidity, and supporting cellular functions (Fig. 5F) (van Meer et al., 2008). Elevated levels of GPC during early lactation have been reported to prevent ketosis and alleviate metabolic stress in dairy cows (Klein et al., 2012). As a marker of fatty acid mobilization, GPC helps balance the negative energy status characteristic of early lactation by compensating for increased energy demands (Klein et al., 2012). Therefore, supplementation with YC enriched with high levels of bioactive compounds (e.g., choline and GPC) may enhance neonatal cognitive function, promote brain development, support rapid cellular proliferation, and mitigate metabolic stress in newborn mammals.

Metabolites in Cluster 4, comprising 330 compounds, exhibit a progressive and continuous decrease from YC through YT to YM (Fig. 5G and Table S5). Compared to Cluster 3, KEGG pathway enrichment analysis of Cluster 4 revealed not only pathways related to immune regulation (e.g., asthma) and phospholipid metabolism (e.g., choline metabolism in cancer and GPL metabolism), but also those associated with branched-chain amino acids (BCAAs) biosynthesis, specifically valine, leucine, and isoleucine biosynthesis, which were significantly enriched (Fig. S4D). BCAAs play critical roles in lipogenesis and lipid metabolic regulation. Notably, dietary supplementation with BCAAs in sows has been shown to increase litter birth weight and postnatal growth, while enhancing colostrum fat content by 27.3–35.8 % (Fig. 5H) (Ma et al., 2020). Among *N*-acetylated carbohydrate metabolites, *N*-acetyl-D-lactosamine (LacNAc) exhibited the highest concentration in YC. LacNAc, composed of galactose and *N*-acetylglucosamine, acts as a key bifidogenic factor by modulating the gut microbiota, thereby enhancing immune function and metabolic activity (Garrido et al., 2012). Additionally, LacNAc is an essential structural unit of human milk oligosaccharides (HMOs), which undergo further modifications, such as fucosylation and sialylation, generating a diverse repertoire of terminal glycan structures (Bode, 2006). As a result, LacNAc shows a strong correlation with *N*-acetylneuraminic acid (Neu5Ac), highlighting the coordinated roles of YC in neonatal nutrition and immune support (Fig. 5H). At birth, the circadian rhythm in neonates is not yet fully developed. This is primarily reflected in irregular sleep–wake cycles and unstable melatonin secretion patterns. This is primarily attributed to the immaturity of central circadian regulators, namely the suprachiasmatic nucleus in the hypothalamus and the pineal gland (Rivkees, 2003). Recent studies have shown that the composition of breast milk changes across the 24 h cycle. In particular, the concentration of Trp is higher at night (Akanalçı & Bilici, 2024). Its downstream metabolites, including 5-HT and 5-methoxytryptamine (melatonin), are also significantly elevated in nocturnal breast milk. Mechanistically, although the neonatal pineal gland is not fully mature at birth, it is highly sensitive to exogenous inputs of Trp and 5-HT [Internet] D. A. L. D. L., 2006; Akanalçı & Bilici, 2024; Cubero et al., 2005). These compounds provide substrates for melatonin synthesis, activate serotonin receptors, promote neural circuit maturation, and induce early expression of key melatonin-synthesizing enzymes such as arylalkylamine *N*-acetyltransferase (AANAT) and hydroxyindole-*O*-methyltransferase (HIOMT) ([Internet] D. A. L. D. L., 2006; Akanalçı & Bilici, 2024; Buniyaadi et al., 2025). Together, these processes contribute to the initial establishment of circadian rhythms in neonates. In the present study, *N*-acetylserotonin (NAS), the immediate precursor of melatonin, was found to be most abundant in YC (Fig. 5G). This suggests that the 5-HT metabolic pathway during early lactation is predominantly directed toward melatonin synthesis. Given that endogenous melatonin synthesis in neonates is not fully developed at birth, the high level of NAS in YC may serve as an exogenous source of rhythmic cues, potentially supporting the establishment of circadian rhythm in early life. This metabolite profile mirrors the diurnal variation observed in human breast milk. Importantly, the natural abundance of NAS in YC and YT provides novel evidences supporting the use of yak milk as a promising alternative source of lipids and functional bioactives in infant formula, particularly those aimed at mimicking the neurodevelopmental and chronobiological functions of human breast milk. This finding adds to the growing body of evidence supporting yak milk as a functionally enriched dairy resource for pediatric nutrition.

**Fig. 6:**
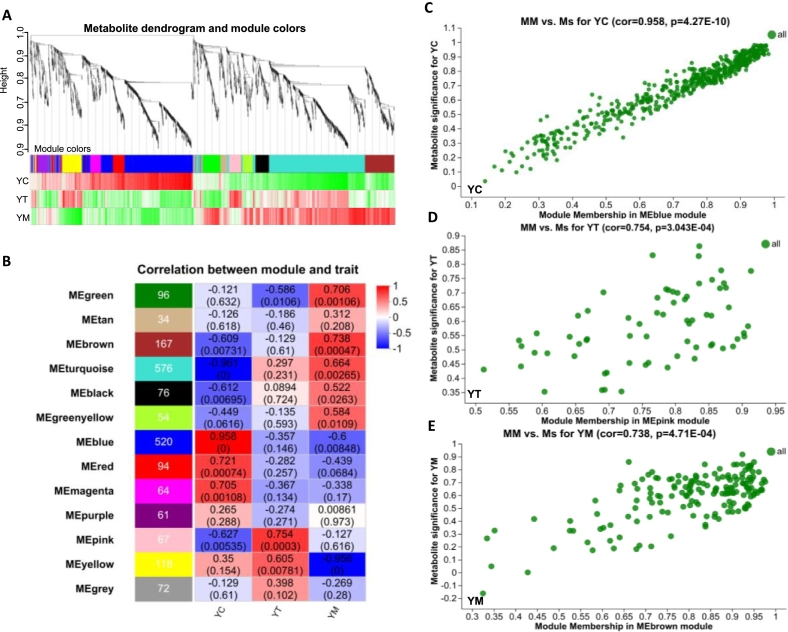
Weighted gene co-expression network analysis (WGCNA) to develop key metabolites in yaks during different lactation periods. (A) Construct a weighted co-expression network utilizing the chosen power values (10). Based on the expression trends of the metabolites, they are divided into modules, where each branch represents a metabolite and each colour represents a module. The bottom presents a heat map showing the correlation between metabolites within each module and phenotypes. The colour represents the strength of the correlation, with red indicating a positive correlation and green indicating a negative correlation. (B) The correlation heatmap of correlation between module and trait (YC, YT and YM). (C-E) There is a highly significant correlation between Metabolite significance (Ms) vs. Module Membership (MM) in this module (*p* < 0.05). (C) The scatterplot of Ms. for YC vs. MM in the blue module. (D) The scatterplot of Ms. for YT vs. MM in the pink module. (E) The scatterplot of Ms. for YM vs. MM in the brown module. (For interpretation of the references to colour in this figure legend, the reader is referred to the web version of this article.)

To further characterize the dynamic changes in metabolite profiles across YC, YT, and YM, weighted gene co-expression network analysis (WGCNA) was performed. An adjacency matrix was first constructed using a soft-thresholding power of 10 to ensure scale-free network topology (Fig. S5A and B). A weighted co-expression network was then constructed, clustering all identified metabolites into 13 distinct modules, each assigned a unique colour (Fig. 6A). Pearson correlation analysis was conducted to assess associations between module eigengenes and lactation stages, with both correlation coefficients (Corr) and *P*-values calculated. The analysis revealed that the blue module was most strongly associated with YC (Corr = 0.958), the pink module with YT (Corr = 0.754), and the brown module with YM (Corr = 0.738) (Fig. 6B). KEGG pathway enrichment analysis of the blue, pink, and brown modules revealed distinct functional profiles (Fig. S5C-E). Furthermore, metabolite significance (MS) versus module membership (MM) analysis was conducted for 520 metabolites in the blue module, 67 in the pink module, and 167 in the brown module (Fig. 6C-E and Table S6). In YC, KEGG enrichment results aligned with previous findings, highlighting pathways related to biomembrane biosynthesis, neuroregulation, and immune responses (e.g., tryptophan metabolism, glycerophospholipid metabolism, sphingolipid signaling pathway, and bile secretion) (Fig. S5C). Notably, oligosaccharides such as 3’-Sialyl-N, 3’-N-Acetyl, and N-Glycolyl derivatives, which are important constituents of HMOs, were enriched in the blue module. These HMOs play a pivotal role in promoting the colonization and proliferation of beneficial gut microbiota, particularly *Bifidobacterium* and *Lactobacillus*, thereby enhancing neonatal gut health and immune function (Fig. 6C) (Bode, 2012). Gut microbiota predominated by *Bifidobacterium* plays an essential role in reducing the incidence of intestinal infections (e.g., necrotizing enterocolitis) and in promoting the development of gut-associated lymphoid tissue (GALT) (Fukuda et al., 2011). This, in turn, facilitates the maturation of the neonatal immune system (Donovan & Comstock, 2016). Oligosaccharides, as integral constituents of glycoproteins and glycolipids, interact with immune cell surface receptors, including sialic acid-binding immunoglobulin-like lectins (Siglecs), to modulate innate immune responses and suppress the overproduction of pro-inflammatory cytokines (Goehring et al., 2016). In particular, Neu5Ac enhances neural signal transduction, thereby supporting neonatal nervous system and cognitive function (Wang, 2009). Collectively, these findings suggest that oligosaccharides abundant in YC may regulate the neonatal immune system through both direct and indirect mechanisms. This effect is potentially mediated through the regulation of gut microbiota composition and associated metabolites, thereby contributing to immune homeostasis. In addition, these bioactive compounds may improve neonatal metabolic health and reduce the long-term risk of developing metabolic syndrome. Furthermore, compared with other lactation stages, YC exhibits the highest concentrations of essential amino acids, including lysine (Lys), Trp, glutamic acid (Glu), proline (Pro), and GPL-associated metabolites. These biomolecules are essential for supporting neonatal growth and development, particularly tissue formation and brain maturation, and also contribute to membrane biosynthesis and immune regulation (Fig. 6C).

In YT, KEGG pathway enrichment analysis identified several significantly enriched biological processes, including oxidative phosphorylation, regulation of lipolysis in adipocytes, circadian entrainment, tryptophan metabolism, and biosynthesis of cofactors (Fig. S5D). As noted earlier, the pentose phosphate pathway plays a pivotal role in enhancing hypoxia tolerance. Ribose-5-phosphate, a key intermediate in the pentose phosphate pathway, was significantly enriched in YT (Fig. 6D). Furthermore, metabolites of the tryptophan-kynurenine pathway (e.g., kynurenine and N′-formylkynurenine) contribute to neonatal health by modulating immune responses and exerting neuroprotective effects (Fig. 6D and E) (Badawy, 2017). In addition, both YT and YM were found to be enriched in a variety of lipid metabolites (e.g., sebacyl-CoA and 2-hydroxybutyric acid), which serve as substrates for fatty acid β-oxidation, generating energy to support neonatal growth and development (Fig. 6D and E). Notably, among fatty acyl metabolites, carnitine not only facilitates β-oxidation but also regulates mitochondrial biogenesis, thereby influencing neonatal metabolic activity and enhancing immune function (Kononov et al., 2022). YM exhibits a substantially higher lipid content, including sphingolipids, phospholipids [e.g., L-α-lysophosphatidylcholine, O-phosphatidylserine, and PS(18:1(9Z)/0:0)], cholesterol, and their corresponding transport and modification enzymes. KEGG pathway enrichment analysis revealed a notable enrichment of the glycerophospholipid metabolism pathway in YM (Fig. S5E). These lipids not only supply energy and essential structural components for cellular membranes, but also play pivotal roles in neurodevelopment and immune regulation (Fig. 6E) (Ballard & Morrow, 2013; Hannun & Obeid, 2018; Zeisel, 2006). Recent studies have confirmed the presence of endogenous cannabinoids in human milk. These bioactive lipids are involved in modulating infant suckling behavior, exerting calming effects, and promoting neural plasticity. This may explain the significant enrichment of the retrograde endocannabinoid signaling pathway observed in YM (Fig. S5E) (Victora et al., 2016).

To further elucidate the metabolic uniqueness of yak milk, we compared our results with recent NMR-based metabolomic profiles of high-altitude-adapted indigenous cows (*Bos indicus*) from the Leh–Ladakh region (Amarjeet et al., 2025). In that study, 46 milk metabolites were identified across colostrum, transitional, and mature milk stages. In both yak and Ladakhi cow colostrum, BCAAs (e.g., valine, leucine, and isoleucine) were markedly elevated and declined progressively throughout lactation. Yak milk is enriched in Lys, Phe, and Trp, which are directly involved in aminoacyl-tRNA biosynthesis and neurodevelopmental pathways (Fig. 4C). Notably, Trp and its metabolite NAS, which play essential roles in circadian rhythm regulation and immune modulation, were exclusively detected in yak milk but were absent in Ladakhi cow milk. Both species showed elevated levels of choline, UDP-galactose and UDP-glucose in colostrum. YC displays distinct metabolic signatures likely associated with more extreme environmental adaptation. Notably, the presence of sialylated oligosaccharides (e.g., 3’-*N*-acetylneuraminyl-*N*-acetyllactosamine), which were not reported in Ladakhi cow milk, suggests an enhanced role in neurodevelopment and intestinal colonization. YC contained significantly higher levels of 3-(3-amino-3-carboxypropyl) uridine and other modified nucleosides, which were absent from the Ladakhi cow milk dataset. While uridine, dCTP, and UDP-sugars were present in both species, yak milk featured higher enrichment in OMP and other pyrimidine pathway intermediates, supporting heightened RNA synthesis and cell proliferation during early lactation. In contrast, mature milk from Ladakhi cows contained higher levels of energy-related metabolites such as creatine phosphate, lactose, and 2-oxoglutarate, supporting sustained energy delivery and mitochondrial activity during later lactation. Similarly, YM showed an enrichment in 3-hydroxyoctanoyl carnitine and TCA intermediates, reinforcing the energetic shift that accompanies milk maturation. Nonetheless, the yak milk metabolome displayed stronger enrichment of immune-supportive oligosaccharides and modified nucleosides (e.g., 3-(3-amino-3-carboxypropyl) Uridine), which were undetected in Ladakhi cow milk.

Both datasets showed significant enrichment in aminoacyl-tRNA biosynthesis, galactose metabolism, and citrate cycle pathways. However, yak milk additionally exhibited activation of tryptophan metabolism, sialic acid biosynthesis, and riboflavin metabolism, suggesting a broader metabolic program oriented toward immune development, neurodevelopment, and oxidative stress resilience. Taken together, the distinct enrichment of sialylated oligosaccharides, tryptophan-derived neuroactive compounds, and lipid-oxidative cofactors in yak milk underscores its unique compositional profile. Many of these components are also found in human milk but are largely absent or present at lower levels in cow milk, highlighting the superior potential of yak milk to support neonatal immune function, neurodevelopment, and hypoxia adaptation in high-altitude environments.

The comprehensive metabolite landscape of YM underscores its multifaceted role in delivering a rich array of lipids, proteins, amino acids, nucleotides, and other bioactive compounds. This metabolic complexity highlights the sustained support that mature milk provides for infant immunity, neurodevelopment, autophagy, and membrane remodeling. As the final and most stable stage of lactation, YM is optimized to meet the prolonged nutritional, immunological, and developmental needs of growing infants. In contrast, YT serves as an intermediate phase bridging the high immunological activity of YC and the enhanced nutritional profile of YM. As such, YT plays a crucial role in energy metabolism, immune modulation, neurodevelopment, and the physiological interplay between mother and offspring.

Although our untargeted metabolomics analysis covered a broad range of metabolites, the detection and accurate identification of certain low-abundance bioactive compounds known to exist in human breast milk may have been limited by the sensitivity threshold of the UHPLC-Q Exactive HF-X platform. While these substances may be present in yak milk at trace levels, they were not reliably detected or quantified under the current analytical conditions.

## 4 Conclusions

In this study, a comprehensive untargeted metabolomics approach was employed to elucidate the dynamic metabolic reprogramming and functional specialization of YC, YT, and YM across distinct lactation stages. The findings revealed that oligosaccharide metabolites (e.g., LacNAc, 3’-Sialyl-N-Acetylneuraminic acid and N-Glycolylneuraminic acidenriched) in YC promote the proliferation of beneficial gut microbiota, thereby modulating neonatal immune responses and supporting early neurodevelopment. In addition, choline and GPC were observed to enhance biomembrane synthesis in neonates and to exert protective effects against ketosis and metabolic stress in calves. YT exhibited distinct metabolic features by balancing neonatal immune requirements with increasing energy demands. Notably, Trp and NAS, enriched in both YC and YT, were implicated in circadian rhythm regulation. Furthermore, the accumulation of arginine and pentose phosphate pathway intermediates during these lactation stages is hypothesized to enhance hypoxia tolerance and support neurocognitive development in neonatal calves raised in high-altitude, low-oxygen environments. Overall, this study provides novel insights into stage-specific metabolic reprogramming in yak milk and offers a scientific foundation for the development of yak-derived functional dairy products and the optimization of infant formula.

## CRediT authorship contribution statement

**Yunduan Wang:** Writing – original draft, Methodology, Investigation. **Daoliang Lan:** Supervision, Funding acquisition, Conceptualization. **Shaohui Beng:** Methodology, Data curation. **Shunyang Liu:** Formal analysis, Data curation. **Xueer Mu:** Formal analysis, Data curation. **Kechao Chen:** Validation, Methodology, Investigation. **Yueyue Li:** Formal analysis, Data curation. **Meng Xu:** Validation, Methodology, Investigation. **Jian Li:** Supervision, Resources, Funding acquisition, Data curation. **Wei Fu:** Writing – review & editing, Project administration, Funding acquisition, Conceptualization.

## Declaration of competing interest

The authors declare that they have no known competing financial interests or personal relationships that could have appeared to influence the work reported in this paper.

## Data Availability

No data was used for the research described in the article.

## Appendix A Supplementary data

Supplementary material 1 Image 1

Supplementary material 2 Image 2

Supplementary material 3 Image 3

Supplementary material 4 Image 4

Supplementary material 5 Image 5

Supplementary material 6 Image 6

Supplementary material 7 Image 7
