# Supplementary material for: Untargeted metabolomics reveals stage-specific metabolic signatures in yak colostrum, transitional milk and mature milk
Source: Food Chem X. 2025 Jun 2;28:102614. doi: 10.1016/j.fochx.2025.102614 (PMC12181027; doi:10.1016/j.fochx.2025.102614)
Supplement: Supplementary file 1 — Supplementary material 1 [file mmc1.docx]

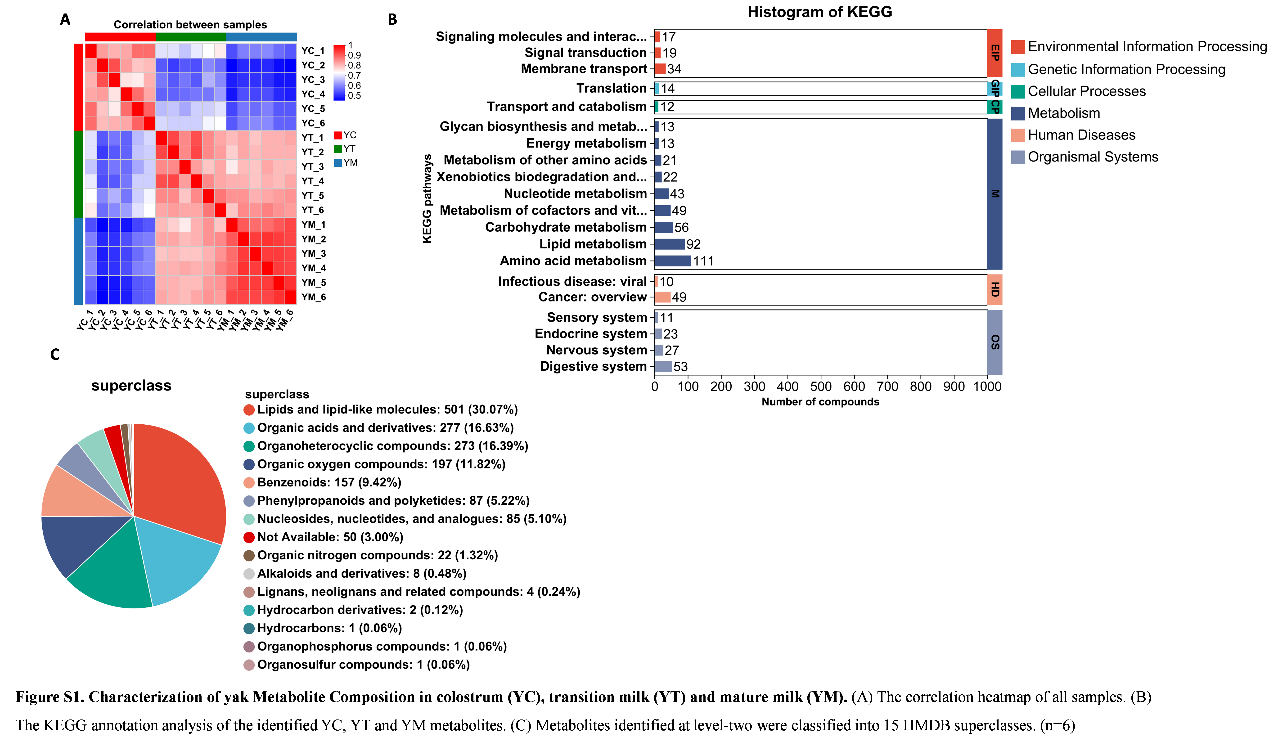


**Figure S1. Characterization of the Metabolite Composition in yak colostrum (YC), transition milk (YT) and mature milk (YM).** (A) The correlation heatmap of all samples. (B) The KEGG annotation analysis of the identified metabolites in YC, YT and YM. (C) Classification of the identified metabolites in YC, YT and YM. *n* = 6.


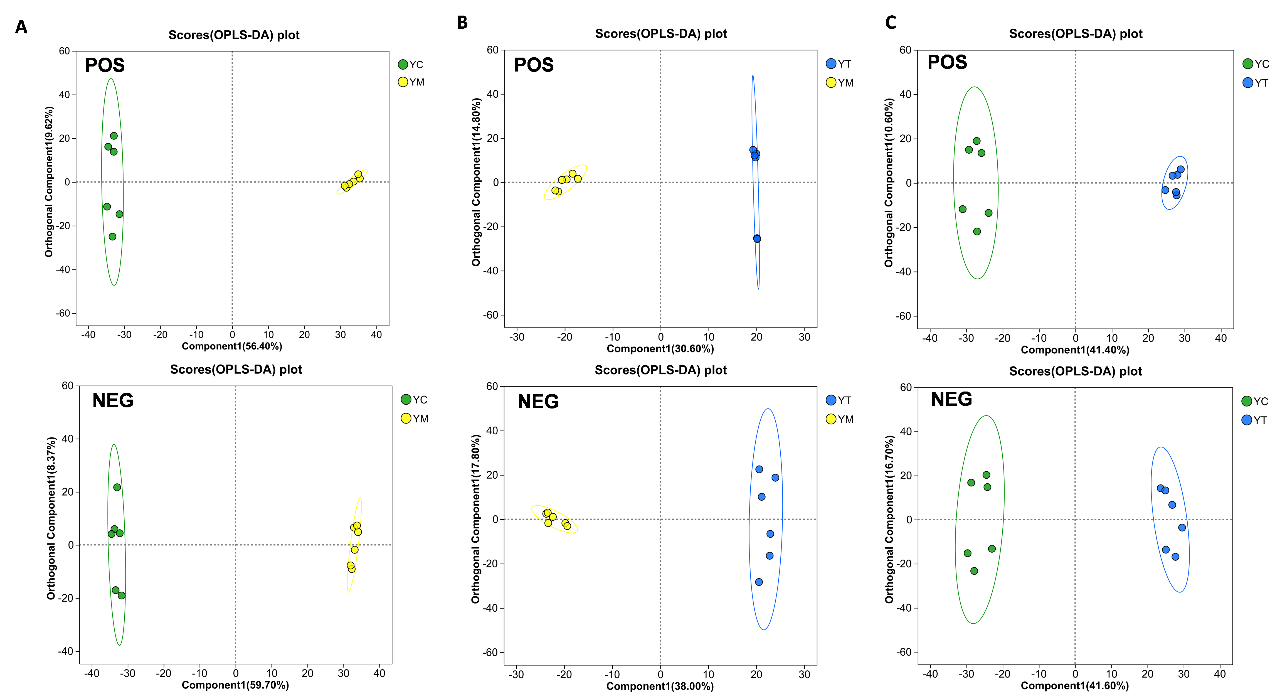


**Figure S2. Orthogonal Partial Least Squares Discriminant Analysis (OPLS-DA) under the POS and NEG model**. (A) YC vs. YM. (B) YT vs. YM. (C) YC vs. YT.


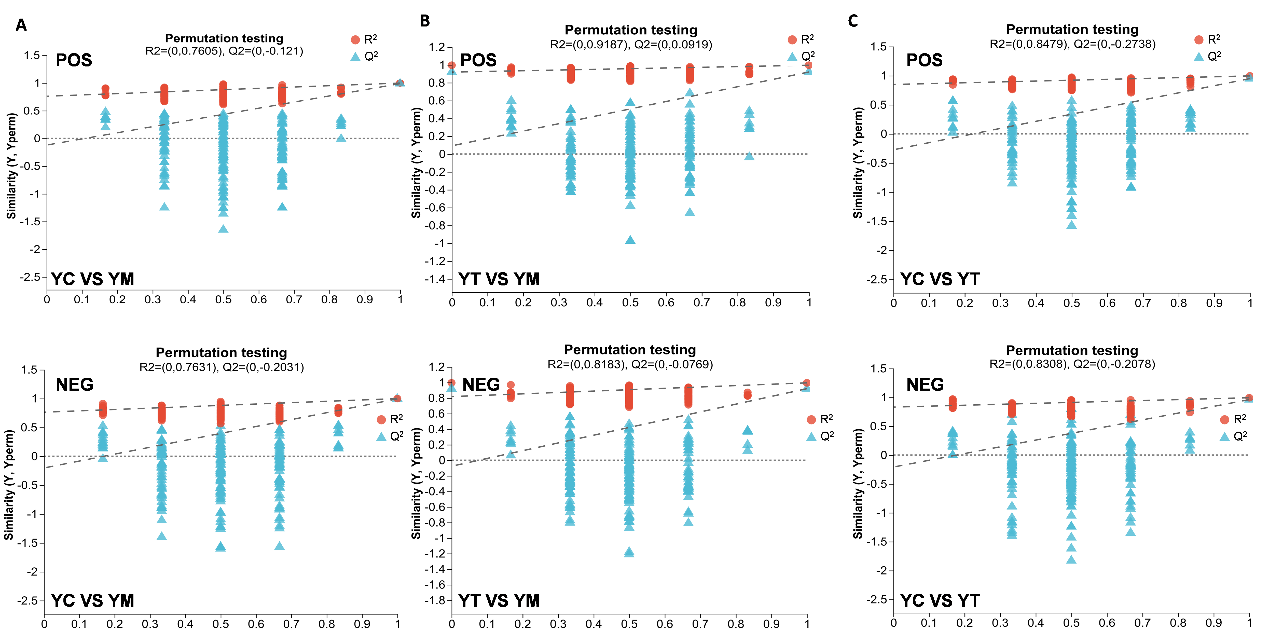


**Figure S3. Permutation test plots for** yak YC vs. YM (A), YT vs. YM (B), and YC vs. YT (C) analyzed under the POS and NEG model (*200* times).


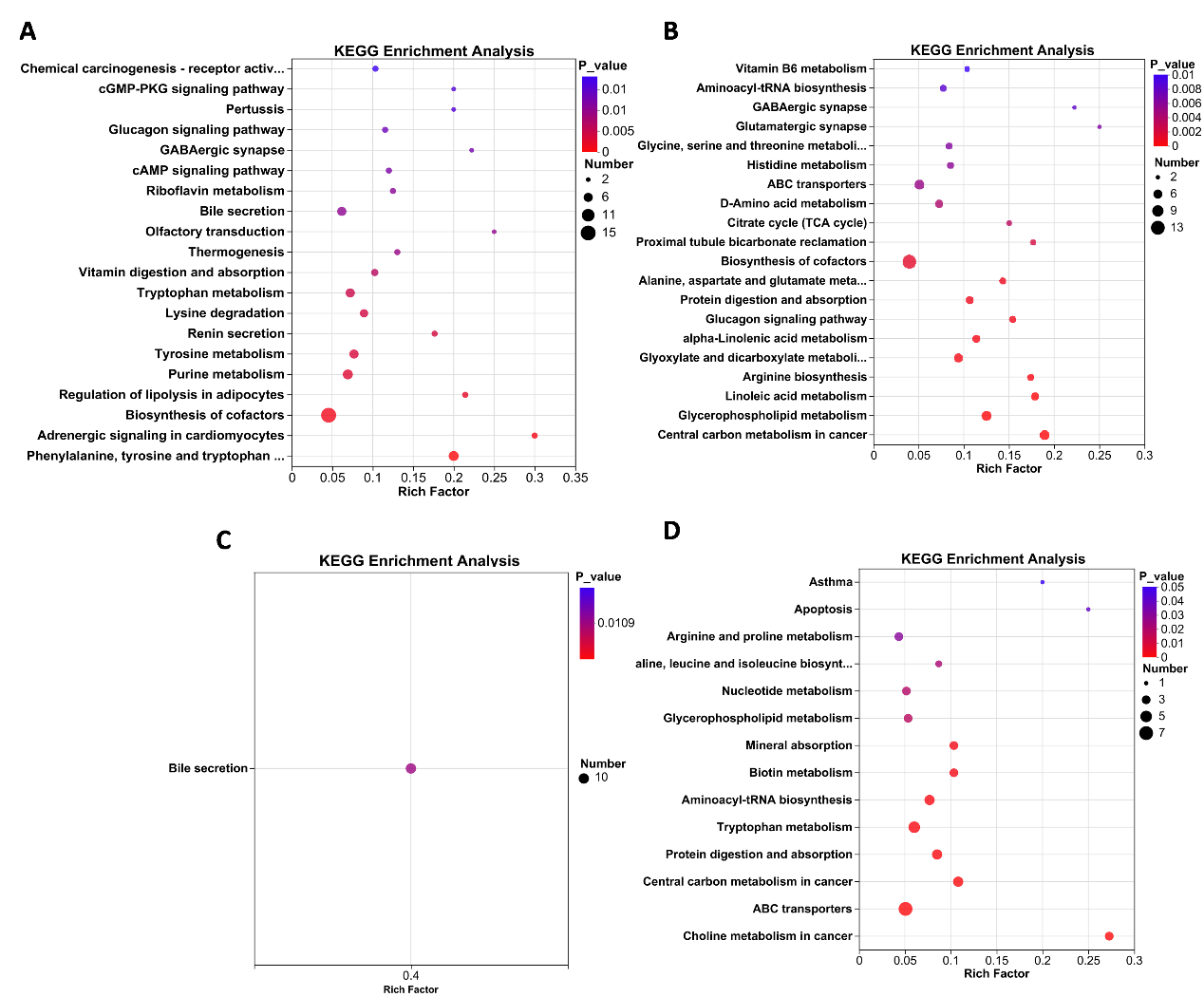


**Figure S4. Enriched KEGG pathway (*p* < 0.05) analysis of the metabolites in** cluster1 (A), cluster2 (B), cluster3 (C) and cluster4 (D).


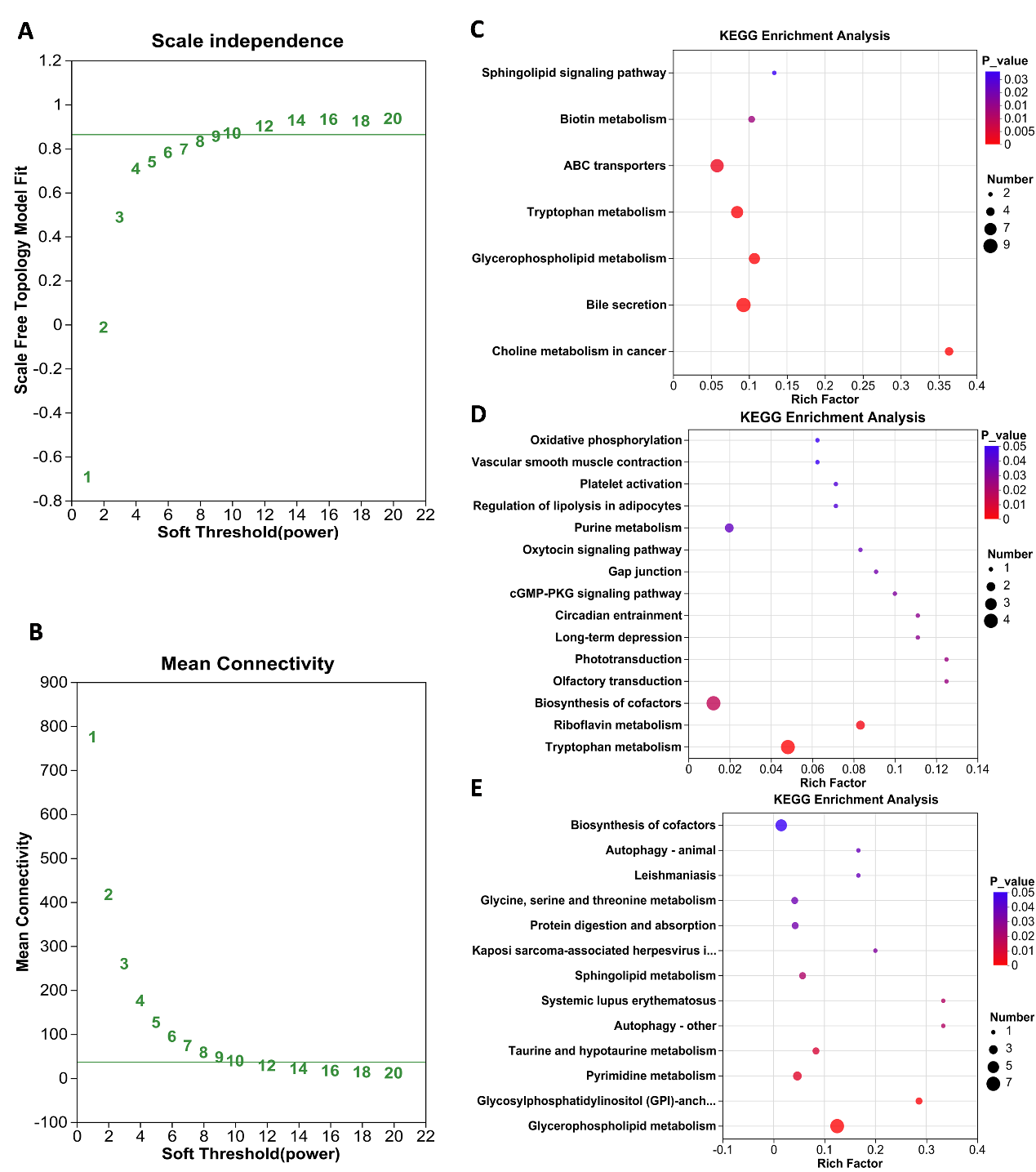


**Figure S5. Weighted gene co-expression network analysis (WGCNA) to develop key metabolites in yaks during different lactation periods.** (A-B) The ideal power for soft-thresholding was determined to be 10. (C-E) Enriched KEGG pathway (*p* < 0.05) analysis of the metabolites in blue module (C), pink module(D) and brown module(E).
